# The AprV5 Subtilase Is Required for the Optimal Processing of All Three Extracellular Serine Proteases from *Dichelobacter nodosus*

**DOI:** 10.1371/journal.pone.0047932

**Published:** 2012-10-24

**Authors:** Xiaoyan Han, Ruth M. Kennan, David L. Steer, A. Ian Smith, James C. Whisstock, Julian I. Rood

**Affiliations:** 1 Australian Research Council Centre of Excellence in Structural and Functional Microbial Genomics, Monash University, Clayton, Victoria, Australia; 2 Department of Microbiology, Monash University, Clayton, Victoria, Australia; 3 Department of Biochemistry and Molecular Biology, Monash University, Clayton, Victoria, Australia; East Carolina University School of Medicine, United States of America

## Abstract

*Dichelobacter nodosus* is the principal causative agent of ovine footrot and its extracellular proteases are major virulence factors. Virulent isolates of *D. nodosus* secrete three subtilisin-like serine proteases: AprV2, AprV5 and BprV. These enzymes are each synthesized as precursor molecules that include a signal (pre-) peptide, a pro-peptide and a C-terminal extension, which are processed to produce the mature active forms. The function of the C-terminal regions of these proteases and the mechanism of protease processing and secretion are unknown. AprV5 contributes to most of the protease activity secreted by *D. nodosus*. To understand the role of the C-terminal extension of AprV5, we constructed a series of C-terminal-deletion mutants in *D. nodosus* by allelic exchange. The proteases present in the resultant mutants and their complemented derivatives were examined by protease zymogram analysis, western blotting and mass spectrometry. The results showed that the C-terminal region of AprV5 is required for the normal expression of protease activity, deletion of this region led to a delay in the processing of these enzymes. *D. nodosus* is an unusual bacterium in that it produces three closely related extracellular serine proteases. We have now shown that one of these enzymes, AprV5, is responsible for its own maturation, and for the optimal cleavage of AprV2 and BprV, to their mature active forms. These studies have increased our understanding of how this important pathogen processes these virulence-associated extracellular proteases and secretes them into its external environment.

## Introduction

*Dichelobacter nodosus* is a slow growing gram-negative anaerobic bacterium that is the principal causative agent of footrot in ruminants. Ovine footrot is characterized by the separation of the keratinous hoof from the underlying tissue, leading to lameness, loss of body weight, and reduced wool growth and quality, which results in significant economic losses [1]. Clinical disease is dependent upon the virulence properties of the *D. nodosus* isolate and the presence of warm wet climatic conditions. There are two major clinical forms of footrot, benign and virulent, with benign footrot presenting as an interdigital dermatitis with no further disease progression whilst virulent disease leads to a severe under-running of the horn of the hoof. The virulence factors of *D. nodosus* include its extracellular subtilisin-like serine proteases (or subtilases) [2]–[6], type IV fimbriae [7], [8] and potentially, the *vrl* and *vap* genomic islands, which are preferentially associated with virulent strains [9]–[12].

Subtilases are produced by a wide variety of archaea, bacteria, fungi and eukaryotes. They mostly are synthesized as pre-pro-enzyme precursors that are subsequently translocated across the cell membrane and then activated by cleavage of the 27–280 residue pro-domains [13]. The relatively conserved catalytic domains contain 268–511 residues, with some members of the subtilase family also having C-terminal extensions with variable lengths and less sequence conservation [13]. The function of the C-terminal extensions is also variable. Studies on aqualysin I from *Thermus aquaticus* YT-1 [14], the serine protease SSP from *Serratia marcescens* [15] and the IgA protease from *Neisseria gonorrhoeae* [16] showed that the C-terminal extensions of these proteins are required for extracellular secretion of the proteases, while the C-terminal extension of the subtilase SISBTS from the tomato plant is required for both pro-domain processing and secretion [17]. By contrast, it has been shown that the C-terminal extension of MCP-03, a subtilase from *Pseudoalteromonas sp.* SM9913, is not crucial for secretion, but instead negatively affects catalytic efficiency and is essential for protease thermostability [18]. Studies on the *Lactococcus lactis* protease suggested that some residues in the C-terminal extension affect catalytic activity [19].

Three closely related subtilases are secreted by virulent strains of *D*. *nodosus*: two acidic proteases, AprV2 and AprV5, and the basic protease BprV. The equivalent proteases in benign strains are AprB2, AprB5 and BprB [20], [21]. AprV5 has been shown to be responsible for most of the extracellular protease activity in a virulent strain of *D. nodosus*, VCS1703A, although AprV2 is responsible for most of the extracellular elastase activity and is essential for virulence [6]. These proteases are encoded by separate genes and are synthesized as precursors with an N-terminal pre-pro-region, a serine protease domain and a C-terminal extension. Based on the cleavage sites determined previously [20], [21], the estimated sizes of the mature domains of AprV5, AprV2 and BprV are 36.0 kDa, 36.4 kDa and 35.8 kDa, respectively; their respective C-terminal extensions are 13.9 kDa, 14.0 kDa and 14.1 kDa in size. The amino acid sequences and structures of the catalytic protease domains are highly conserved (∼65% identity) [6], [22], but the sequences of the C-terminal extensions are less conserved, with only approximately 35% similarity. The C-terminal extension of AprV5 contains a P-domain, which is typically associated with eukaryotic pro-protein convertases that belong to the subtilisin-like superfamily [13], [23]. The active proteases are produced by cleavage of the pre-pro region and the C-terminal extension [2], [24], [25]. The mechanism of protease processing and secretion is unknown, although type IV fimbriae are required for optimal secretion [7], [8], [26].

In this study, we aimed to examine the mechanism of processing of the extracellular proteases from *D. nodosus* and to determine the role of their C-terminal extensions. To achieve these aims we constructed a series of C-terminal truncated *aprV5* mutants in *D. nodosus* by allelic exchange. The results showed that AprV5 was required for efficient processing of AprV2 and BprV and that the C-terminal extension of AprV5 was required for maximal AprV5 activity.

## Materials and Methods

### Strains, Plasmids, and Growth Conditions

Bacterial strains and plasmids are listed in Table 1. *Escherichia coli* DH5α and NovaBlue cells used for plasmid propagation and cloning experiments were grown at 37°C on 2×YT medium [27]. The transformable virulent *D. nodosus* strain VCS1703A and its derivatives were grown in an Anaerobic Chamber (Coy Laboratory Products Inc.) in an atmosphere of 10% (vol/vol) H_2_, 10% (vol/vol) CO_2_, and 80% (vol/vol) N_2_ on Eugon (BBL) yeast extract (EYE) agar with 5% (vol/vol) defibrinated horse blood (Bio-lab) or in TAS broth [28], as described previously [7], [26]. When required, media were supplemented with the following antibiotics at the indicated concentrations: for *E. coli*, ampicillin (100 µg/ml), kanamycin (20 µg/ml) or erythromycin (150 µg/ml), and for *D. nodosus*, ampicillin (10 µg/ml), kanamycin (10 µg/ml) or erythromycin (1 µg/ml).

**Table 1:** Bacterial strains and plasmids.

Strain or Plasmid	Characteristics	Source or References
**Strains**		
*** E. coli***		
DH5α	F^−^ *endA1 hsdR17* (r_k_^−^m_k_^−^)*thi-1*λ^−^ *recA1 gyrA96relA1 rhoA supE44 deoRф80dlacZ*ΔM15*Δ(lacZYA argF*)U169	Invitrogen
NovaBlue	*endA1 hsdR17* (r_K12_^–^ m_K12_^+^) *supE44 thi-1 recA1 gyrA96 relA1 lac* F′[*proA^+^B^+^ lacI^q^Z*Δ*M15*::Tn*10*] (Tet^R^)	Novagen
*** D. nodosus***		
VCS1703A	*D. nodosus* serogroup G, virulent; wild-type	Egerton, J. (University of Sydney)
JIR3743	VCS1703A*aprV2*Ω*tet*(M) (*aprV2*)	[6]
JIR3756	VCS1703A*aprV5*Ω*bla* (*aprV5*)	[6]
JIR3883	JIR3756*rrnA*Ω*aprV5^+^* (*aprV5/aprV5^+^*)	[6]
JIR3900	JIR3743Δ*tet*(M)Ω*aprV2^+^erm*(B) (*aprV2/aprV2^+^*)	[6]
JIR3928	VCS1703A*bprV*Ω*erm*(B) (*bprV*)	[6]
JIR3930	JIR3928Δ*erm*(B)Ω*bprV^+^*Kan^R^ (*bprV/bprV^+^*)	[6]
JIR3947	VCS1703A*aprV5*Δ*478–595ΩaphA-3* (*aprV5*Δ*C1*)	VCS1703A transformed with pJIR3520
JIR3953	VCS1703A*aprV5*Ω*6xhisaphA-3* (*aprV56xhis*)	VCS1703A transformed with pJIR3661
JIR3956	VCS1703A*aprV5*Δ*503–595ΩaphA-3* (*aprV5*Δ*C2*)	VCS1703A transformed with pJIR3708
JIR3965	JIR3956Δ*aphA-3ΩaprV5^+^erm*(B) (*aprV5*Δ*C2/V5^+^*)	JIR3956 transformed with pJIR3730
JIR3968	JIR3947Δ*aphA-3ΩaprV5^+^erm*(B) (*aprV5*Δ*C1/V5^+^*)	JIR3947 transformed with pJIR3730
JIR3969	VCS1703A*aprV5*Δ*560–595ΩaphA-3* (*aprV5*Δ*C3*)	VCS1703A transformed with pJIR3729
JIR3978	JIR3969Δ*aphA-3ΩaprV5^+^erm*(B) (*aprV5*Δ*C3/V5^+^*)	JIR3969 transformed with pJIR3730
**Plasmids**		
pUC18K	pUC18 *Sma*IΩ*aphA-3*, nonpolar base vector	[30]
pGEM7Zf(−)	Ap^r^, *lacZ^+^* cloning vector	Promega
pJIR3520	pUC18KAsp718/SacIΩ(1.5-kb LHS PCR product including *aprV5*Δ*478–595*) BamHI/XbaIΩ(1.5-kb RHS PCR product containing downstream of *aprV5*)	Recombinant
pJIR3661	pUC18KAsp718Ω(1.2-kb PCR product containing *aprV5* plus penta his) XbaI/PstIΩ(1.2-kb PCR product containing downstream of *aprV5*)	Recombinant
pJIR3695	pGEM7zf(-)KpnIΩ(1.9-kb PCR product containing *aprV5+*)	Recombinant
pJIR3708	pJIR3520Asp718/SacIΔ(1.5-kb LHS PCR product including *aprV5*Δ*478–595*)Ω(1.4-kb PCR product containing *aprV5*Δ*503–595*)	Recombinant
pJIR3711	pUC18KKpnIΩ(1.9-kb fragment containing the *aprV5* C-terminal encoding region) XbaI/PstIΩ(1.2-kb PCR product containing downstream of *aprV5*)	Recombinant
pJIR3729	pUC18KEcoRI/SacIΩ(1.8-kb *aprV5*Δ*560–595* dropped from pJIR3695/SacI/EcoRI) BamHI/XbaIΩ(1.5-kb RHS PCR product containing downstream of *aprV5*)	Recombinant
pJIR3730	pJIR3711SmaIΔ*aphA-3*Ω(*erm*(B) dropped from pJIR2412/EcoRV/SmaI)	Recombinant

### DNA Manipulation and Molecular Techniques

Unless otherwise stated, standard procedures were used [27]. *D. nodosus* genomic DNA was prepared using a QIAGEN DNeasy kit, according to the manufacturer’s instructions. Southern hybridizations were performed as described previously [26], [29], with probes specific for the relevant antibiotic resistance genes or the target genes. Reverse transcriptase (RT)-PCR was carried out as previously described [8]. DNA sequencing was performed using an Applied Biosystems 3730S Genetic Analyser. Sequence data were compiled using Sequencher version 3.0 (Gene Codes Corporation) or Vector NTI advance™ 11 (Invitrogen).

### Construction of Suicide Vectors and Mutants of *D. nodosus*

All of the suicide vectors used in this study were constructed in pUC18K [30], which does not replicate in *D. nodosus*. They contained two 1.2-kb to 1.9-kb *D. nodous*-derived DNA fragments that were located on either side of an *aphA-3* kanamycin-resistance cassette, with a *bla* ampicillin-resistance gene located on the plasmid vector. Therefore, *D. nodosus* transformants that were derived from double crossovers could be obtained by selecting for kanamycin resistance and screening for susceptibility to ampicillin.

To construct an *aprV5*Δ*478–595* mutant, the suicide vector pJIR3520 was constructed as follows. A 1.5-kb PCR product that contained the *aprV5* gene region that encoded AprV5 residues 1–477 was cloned into the SacI/KpnI sites of pUC18K followed by the cloning of a 1.5-kb PCR fragment located downstream of *aprV5* into the BamHI/XbaI sites of the resultant plasmid. To construct the *aprV5*Δ*503–*595 suicide vector pJIR3708, the 1.5-kb *aprV5*Δ*478–595* fragment of pJIR3520 was deleted by SacI/KpnI digestion and replaced with a 1.4-kb PCR product containing *aprV5*Δ*503–595*. The *aprV5*Δ*560–595* suicide vector pJIR3729 was constructed in a similar way. It contained the 1.5-kb *aprV5* downstream region inserted into BamHI/XbaI sites and a 1.8-kb fragment containing *aprV5*Δ*560–*595 that was obtained by EcoRI/SacI digestion from pJIR3695, a plasmid containing a full-length *aprV5* gene cloned into the KpnI site of pGEM7zf(−). Finally, an *aprV5His_6_* vector (pJIR3661) was constructed in a similar manner by cloning a 1.2-kb PCR product containing an N-terminal truncated *aprV5* plus a hexahistidine-encoding sequence into the KpnI site and cloning a 1.2-kb PCR product downstream of *aprV5* into the XbaI/PstI sites of pUC18K.

To complement the truncated *aprV5* mutants, a 1.2-kb PCR product from the region downstream of *aprV5* was cloned into the XbaI/PstI sites of pUC18K and then a 1.9-kb fragment containing the region encoding AprV5 C-terminal extension was cloned into the KpnI site of the resultant plasmid to form pJIR3711. To enable an antibiotic selection for the complemented strains, *aphA-3* was deleted from pJIR3711 by SmaI digestion and replaced by an *erm*(B) cassette from pJIR2412 [26] to form the complementation vector pJIR3730. The complemented strains were selected on erythromycin and screened for susceptibility to kanamycin and ampicillin.

Plasmid DNA was introduced into *D. nodosus* by natural transformation and mutants were constructed by homologous recombination between the double crossover suicide vectors and the *D. nodosus* chromosome, as described previously [7], [8]. Transformants were initially screened by their resistance or susceptibility to the appropriate antibiotics as described above. Potential mutants were analysed by PCR using relevant antibiotic-resistance gene and target gene primers. Southern hybridization was used to confirm that the C-terminal extension encoding-region had been deleted or that a 6xHis-encoding tag had been incorporated onto the C-terminus of *aprV5* by double crossover events.

### SDS-polyacrylamide Gel Electrophoresis (SDS-PAGE) and Immunoblotting

Whole cell extracts of *D. nodosus* strains were prepared and culture supernatants were trichloroacetic acid (TCA)-precipitated as previously described [8]. Proteins were detected by SDS-PAGE on 4–15% gradient gels (BioRad) after staining with Coomassie Brilliant Blue. The proteases AprV5 and BprV were analysed by immunoblotting with a 1∶4000 diluted AprV5- and BprV-specific antisera raised in sheep (O. Dhungyel & R. Whittington, University of Sydney), respectively. AprV2 was immunoblotted with a 1∶300 dilution of AprV2-specific rabbit antisera (Millipore).

### Extracellular Protease Assays

Caseinase activity was detected qualitatively by growing *D. nodosus* colonies on EYE agar containing 2% (wt/vol) skim milk [31]. Total protease activity in the culture supernatant was determined quantitatively using azocasein (Sigma) as the substrate, as previously described [7]. One unit of protease activity is defined as the amount of enzyme required to digest 1 µg of azocasein per min [7]. Protease zymograms were carried out as previously described [32], except that they were run on a BioRad Mini-Protean RIII apparatus. *D. nodosus* culture supernatant (10 µl) was mixed with 5 µl of sample buffer (50% (wt/vol) sucrose, 0.1% (wt/vol) bromophenol blue) and subjected to electrophoresis at 100 V for approximately 2 h, until the dye reached the bottom of the gel. After electrophoresis, the polyacrylamide gel was placed on a glass plate, overlaid with gelatin/agarose and incubated for 75 min at 37°C in a moist chamber. The gelatin/agarose gel was removed and developed by submerging it in saturated ammonium sulphate solution that had been heated to approximately 60°C. The gel was washed in tap water prior to photography.

### Mass Spectrometry and Protein Identification

Protein bands of interest were excised from Coomassie Brilliant Blue stained gels and prepared for MS analysis as described previously [33]. After the washing and dehydration steps the gel pieces were rehydrated with a solution containing 0.5 µg of sequencing grade trypsin, incubated at 37°C overnight and sonicated, Digested samples were co-spotted onto the target plate with matrix solution (10 mg/ml of α-cyano-4-hydroxycinnamic acid) in 50% acetonitrile-0.1% TFA) and analysed on an Applied Biosystems 4700 Proteomics Analyser MALDI TOF/TOF in reflectron mode with a mass range of 800 to 3500 Da, with a focus mass of 1400 Da at 1500 shots per spectra. The 4700 Series Explorer software automatically selected the 15 most intense peptides as precursor masses for MS/MS analysis, acquired in the order of decreasing intensity. MS/MS analysis was carried out in reflectron mode with a relative precursor mass window of 50 resolution with metastable ion suppression on and spectra summed at 2500 shots/spectrum.

PMF and MSMS data were compiled by GPS explorer software Ver. 3 (build 311) (Applied Biosystems) and searched using the MASCOT search engine (version 1.9, Matrix Science Inc.,) against an in-house database compiling the FASTA formatted *Dichelobacter nodosus* genome obtained from the National Centre for Biotechnology Information (NCBI) FTP site. The following search parameters were used: missed cleavages, 1; peptide mass tolerance, ±50 ppm; peptide fragment tolerance, ±0.1 Da; peptide charge, 1+; Variable modification, oxidation (Methionine). Positive matches were accepted based on the protein score above a significance threshold as defined by MASCOT (www.matrixscience.com). Identifications below this threshold were manually verified by interpretation of the MSMS spectra of matched peptides and matched MS peaks.

## Results

### The C-terminal Extension of AprV5 is Required for Processing of AprV2

**Figure 1:**
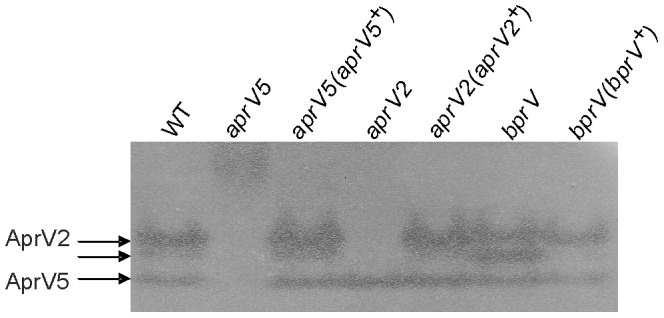
Zymogram analysis of extracellular protease activity. Gelatin was used as a substrate overlay of supernatants separated by native polyacrylamide gel electrophoresis [32] to screen AprV5 and AprV2 activity in 25 h TAS broth cultures from the wild-type strain VCS1703A (WT), the *aprV5* mutant JIR3756 (*aprV5*), the *aprV2* mutant JIR3743 (*aprV2*), the *bprV* mutant JIR3928 (*bprV*) and the complemented derivatives JIR3883 (*aprV5*(*aprV5^+^*)), JIR3900 (*aprV2*(*aprV2^+^*)) and JIR3930 (*bprV*(*bprV^+^*)), respectively.

In studies aimed at determining the role of these extracellular proteases in virulence we previously constructed mutations in each of the three *D. nodosus* extracellular protease genes [6]. To determine if mutation of any of these proteases altered the production of the other two enzymes the extracellular protease activity of these insertionally-inactivated *aprV5*, *aprV2* and *bprV* mutants and their complemented derivatives was characterized by protease zymogram analysis after native gel electrophoresis. The results (Figure 1) showed that, as expected, in the *aprV2* mutant there was no AprV2 activity, as represented by the two missing isoenzyme activity bands; these bands were restored by complementation. Mutation of *aprV2* had no effect on AprV5 activity (the band of highest electrophoretic mobility). By contrast, in the *aprV5* mutant the AprV5 band was absent as expected and the two AprV2 bands were either not present (Figure 1), or in some preparations were very greatly reduced in intensity; all of these protease bands were restored in the complemented *aprV5* derivative (Figure 1). Based on these studies we postulated that AprV5 was required for the correct processing of AprV2. Finally, there was no difference in the active AprV2 or AprV5 bands in the *bprV* mutant (Figure 1). However, an additional protease activity band, of slightly greater electrophoretic mobility than the faster of the AprV2 bands, was observed in this strain. This band disappeared in the *bprV-*complemented derivative, but although difficult to see in this specific preparation; both of the AprV2-derived protease bands were always present in supernatants from this strain. Note that the basic protease BprV can not be observed by this method, as a result of its basic pI. Attempts to visualise BprV activity by carrying out zymogram analysis under different electrophoretic conditions were unsuccessful.

**Figure 2:**
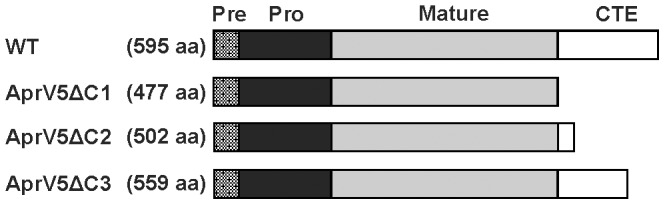
Schematic representation of the C-terminal deleted AprV5 derivatives. The number of amino acid residues in each derivative is indicated as are the pre-, pro-, mature and C-terminal extension (CTE) regions.

To determine if the C-terminal domain of AprV5 was involved in the processing of AprV2 we again used a genetic approach. The *aprV5* sequence encoding the previously determined C-terminal extension, residues 478–595 [25], was deleted by allelic exchange between the suicide vector pJIR3520 and the wild-type chromosome. The genotype of the C-terminal-truncated strain *aprV5*Δ*478–595*, which was designated as *aprV5*Δ*C1* (Figure 2), was confirmed by PCR analysis and Southern hybridization. To ensure that the C-terminal deletion had no effect on protease gene expression, RT-PCR analysis using *aprV5*-, *aprV2*- and *bprV*-specific primers was performed. The results showed that *aprV5*, *aprV2* and *bprV* were all transcribed in this mutant (data not shown). We subsequently constructed two additional AprV5-C-terminal truncated strains, *aprV5*Δ*503–595* (*aprV5*Δ*C2*) and *aprV5*Δ*560–595* (*aprV5*Δ*C3*) (Figure 2), which also were confirmed by PCR and Southern hybridization. All three mutants were complemented by using homologous recombination to reconstitute the deleted C-terminal-encoding *aprV5* regions.

**Figure 3:**
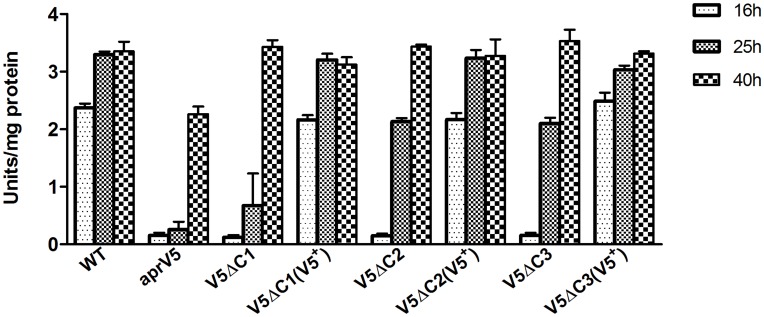
Quantitative analysis of protease activity of deletion mutants. Total protease activity in culture supernatants was determined with azocasein as the substrate. The culture supernatants from 16 h, 25 h and 40 h TAS broth cultures of the wild type strain VCS1703A (WT), the *aprV5* mutant JIR3756 (*aprV5*), the *aprV5*Δ*478–595* strain JIR3947 (*V5*Δ*C1*), the *aprV5*Δ*503–595* strain JIR3956 (*V5*Δ*C2*), the *aprV5*Δ*560–595* strain JIR3969 (*V5*Δ*C3*) were analysed as well as their complemented derivatives: JIR3968 (*V5*Δ*C1*(*V5^+^*)), JIR3965 (*V5*Δ*C2*(*V5^+^*)) and JIR3978 (*V5*Δ*C3*(*V5^+^*)). All values were obtained from three independent biological samples. Error bars represent SEM.

Qualitative protease analysis on skim-milk agar was carried out on the C-terminal deletion strains and their complemented derivatives. The results showed that after 24 h incubation, all of the *aprV5*Δ*C* mutants had reduced protease activity compared to the wild-type strain; this activity was restored in each of the complemented derivatives (data not shown). These results were confirmed by quantitative protease assays carried out on culture supernatants of 16 h (mid-logarithmic growth phase), 25 h (late logarithmic/early stationary phase) and 40 h (stationary phase) TAS broth cultures of each strain, using azocasein as the substrate. An *aprV5* null mutant [6] was included as a negative control in this experiment. The 16 h and 25 h samples, which represented late logarithmic and stationary growth phases, respectively, of each of the *aprV5*Δ*C* mutants, had significantly lower levels (p<0.05; student’s *t-test*) of extracellular protease activity than the wild-type strain (Figure 3). There was no significant difference in activity in the 16 h culture supernatants between any of the mutants, although they all had significantly less protease activity than the wild type (p<0.05). However, the protease activity in 25 h culture supernatants of the *aprV5* and *aprV5*Δ*C1* mutants was significantly lower (p<0.05) than that of the *aprV5*Δ*C2* and *aprV5*Δ*C3* mutants. By contrast, at 40 h there was no significant difference between the *aprV5*Δ*C* mutants and the wild-type strain. The reduced protease activity of 16 h and 25 h cultures of each *aprV5*Δ*C* strain was restored to the wild-type level upon reconstitution (Figure 3). These results implied that deletion of the C-terminal domain of AprV5 delays the appearance of fully functional extracellular protease in the culture supernatant of *D. nodosus*.

**Figure 4:**
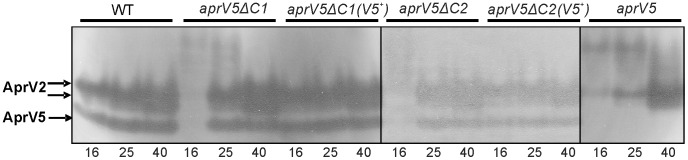
Extracellular protease zymograms of the *aprV5* C-terminal deletion strains. Cultures from 16 h, 25 h and 40 h TAS broth cultures of the wild-type strain VCS1703A (WT), the *aprV5*Δ*478–595* strain JIR3947 (*aprV5*Δ*C1*) and its complemented derivative JIR3968 (*aprV5*Δ*C1*(*V5^+^*)), the *aprV5*Δ*503–595* strain JIR3956 (*aprV5*Δ*C2*) and its complemented derivative JIR3965 (*aprV5*Δ*C2*(*V5^+^*)) and the *aprV5* mutant JIR3756 (*aprV5*) were examined. The profile of the *aprV5*Δ*560–595* strain JIR3969 (*aprV5*Δ*C3*) is not shown but was identical to that of *aprV5*Δ*C2*.

To determine if the C-terminal deletions of AprV5 affected the processing of AprV2 in a similar manner to the *aprV5* null mutant (Figure 1), protease zymogram analysis was carried out. The results showed that the AprV5 activity in the 16 h supernatants from each *aprV5*Δ*C* mutant was significantly reduced (Figure 4); moreover, AprV2 activity also was reduced in these samples. In addition, extra bands of lower electrophoretic mobility than AprV2 were present in these samples, presumably unprocessed forms of the proteases. At 25 h the AprV5 and AprV2 activity bands in the *aprV5*Δ*C* mutants were not discernibly different to those of the wild-type strain (Figure 4), although in the *aprV5*Δ*C1* and *aprV5* null mutant, the extra lower mobility bands were still present, which suggested that there was a delayed processing of proteases in these two mutants. Note that in each of the mutants all of these effects were reversed when the wild-type *aprV5* gene was reconstituted.

### AprV5 is Required for Optimal Processing of AprV2 and BprV

**Figure 5:**
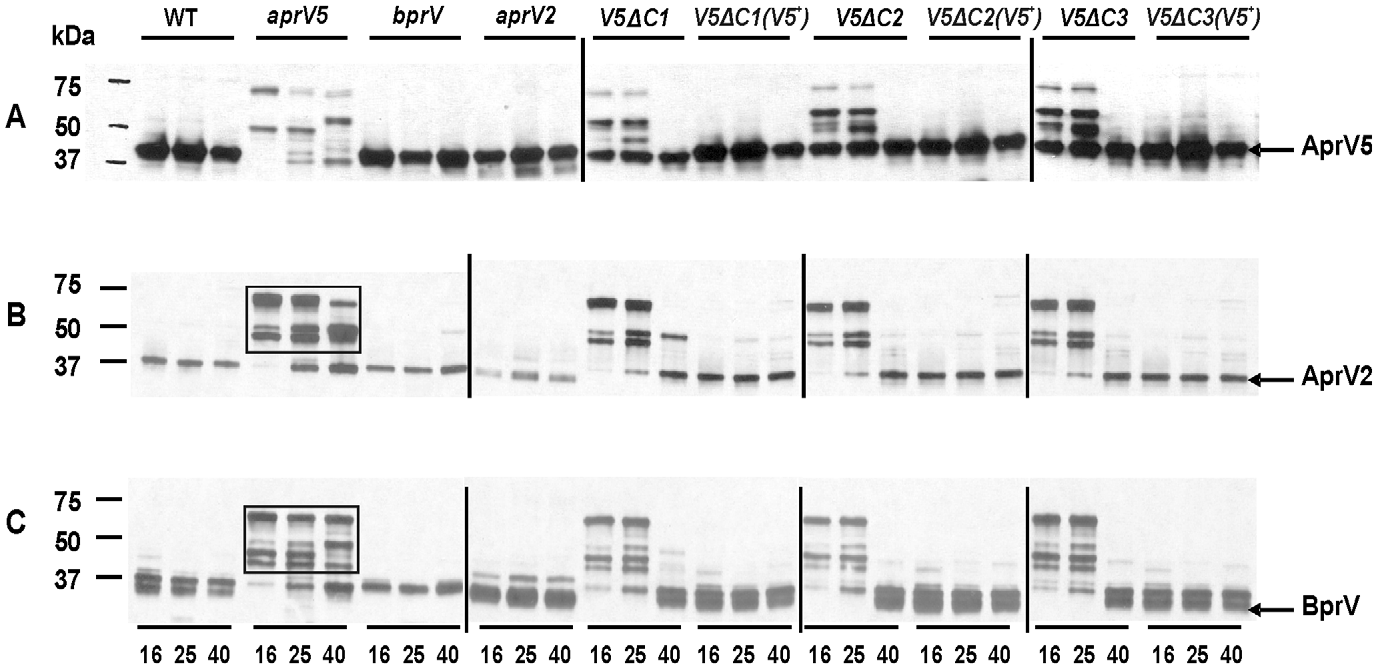
Western immunoblotting of wild-type and mutants with specific protease antisera. Concentrated culture supernatants (3 µg) from 16 h, 25 h and 40 h (from left to right ) TAS growth of the wild type strain VCS1703A (WT), the *aprV5*Δ*478–595* strain JIR3947 (*V5*Δ*C1*), the *aprV5*Δ*503–595* strain JIR3956 (*V5*Δ*C2*), the *aprV5*Δ*560–595* strain JIR3969 (*V5*Δ*C3*) and their complemented derivatives of JIR3968 (*V5*Δ*C1(V5^+^*), JIR3965 (*V5*Δ*C2(V5^+^*) and JIR3978 (*V5*Δ*C3(V5^+^*) were separated on 4–15% gradient SDS-PAGE and analysed by Western immunoblotting with (**A**) AprV5 (1∶4000)-, (**B**) AprV2 (1∶300)- and (**C**) BprV (1∶4000)- antisera. The *aprV5* mutant JIR3756 (*aprV5*), the *aprV2* mutant JIR3743 (*aprV2*) and the *bprV* mutant JIR3928 (*bprV*) were included as controls. The two groups of larger proteins in the *aprV5* preparation are boxed.

To further examine the effect of the C-terminal extension of AprV5 on each of the three individual proteases, equal amounts of the concentrated supernatants from the *aprV5*Δ*C* mutants were analysed on 4–15% gradient SDS-PAGE gels and blotted with specific antisera (Figure 5). Note that in these experiments the results were complicated by unavoidable immunological cross-reaction between these closely related proteases. Accordingly, the individual *aprV5, aprV2* and *bprV* null mutants isolated previously [6] were used as negative controls alongside the wild-type positive control.

Western immunoblotting with AprV5-specific antisera showed that the mature AprV5 band produced by the *aprV5*Δ*C1* mutant was slightly smaller in size than the equivalent band in the wild type. This band was missing in the *aprV5* null mutant. By contrast, the AprV5 band in the *aprV5*Δ*C2* and *aprV5*Δ*C3* mutants was the same size (approx. 40 kDa) as that in the wild-type strain (Figure 5a). These results indicated that the AprV5 C-terminal extension cleavage site was located between residues 478–502, not at residue 478 as previously suggested [25]. In an attempt to determine the C-terminal cleavage site of AprV5, we introduced codons encoding a 6xHis tag onto the 3′-end of *aprV5* in *D. nodosus* by allelic exchange, with the objective of carrying out N-terminal sequence analysis on the cleaved C-terminal extension-6xHis fragment. The resultant *aprV5_His6_* strain (JIR3953) was confirmed as before and protease analysis on skim-milk agar showed that introduction of the 6xHis tag did not affect overall protease production (data not shown). However, Western immunoblotting using 6xHis antibody could not detect a 6xHis-tagged protein from either culture supernatants or whole cell extracts after repeated attempts, suggesting that the C-terminal extension of AprV5 was degraded after protease processing.

The results also showed that in the *aprV5*Δ*C2* and *aprV5*Δ*C3* strains there was a subtle reduction of the amount of mature AprV5 at 16 h and 25 h, compared to the wild-type strain and the complemented derivatives. In addition, there were protein bands of higher molecular size (approx. 50 kDa) that specifically reacted with the AprV5 antisera. We postulated that these bands represented pro-AprV5, which was supported by the observation that in *aprV5*Δ*C1*, these proteins were smaller in size than in *aprV5*Δ*C2* and *aprV5*Δ*C3*, which was consistent with the fact that the mature AprV5 band in this strain was smaller in size. Note that all of the complemented derivatives showed a similar profile to the wild-type strain and that at 40 h all of the deletion mutants had a wild-type profile, with no larger immunoreactive bands. These data support the hypothesis that the C-terminal extension of AprV5 is required for efficient protease processing. Loss of the C-terminal extension delays processing to the mature form.

Western immunoblotting using AprV2-specific antiserum confirmed that AprV5 had a role in the maturation of AprV2. The results showed that the amount of the mature AprV2 band, which migrated at approx. 38 kDa, was greatly reduced in the 16 h and 25 h cultures of the *aprV5*, *aprV5*Δ*C1*, *aprV5*Δ*C2*, and *aprV5*Δ*C3* mutants and that two groups of larger proteins (approx. 65 kDa and 50 kDa) were present in these samples (Figure 5b), these were presumably less processed forms of AprV2. Similar results were obtained when BrpV-specific antiserum was used, with less processed forms of BprV also detected (Figure 5c). Once more, reconstitution of the intact *aprV5* gene reversed this effect and no effects on protease processing were observed at 40 h. These data provided clear evidence that AprV5 was involved in the processing of both AprV2 and BprV. In the *aprV2* and *bprV* mutants there was no reduction in the mature forms of the other two proteases or any evidence for the accumulation of larger proteins (Figure 5a, b & c). Therefore, it appeared highly unlikely that AprV2 and BprV processed each other or AprV5. Western immunoblotting also was carried out on whole cell lysates of each strain using AprV2 and BprV antisera. The results showed that there were no AprV2 or BprV bands present in the whole cell lysates (data not shown), confirming that AprV2 and BprV are processed after secretion.

To confirm that the novel proteins accumulated in the *aprV5* mutants were unprocessed forms of AprV2 and BprV MALDI-TOF-TOF (MS-MS) analysis was performed on in-gel proteins excised from the *aprV5*, *aprV5*Δ*C1* and *aprV5*Δ*C3* lanes. The results (Table 2) revealed that in the extracted 65 kDa bands both pro-AprV2-CTE and pro-BprV-CTE were identified, whereas the 50 kDa bands contained AprV2-CTE and BprV-CTE. Unfortunately, no unprocessed AprV5-derived peptides were detected, despite repeated analyses.

**Table 2:** Identification of unprocessed proteases in the *aprV5* mutant and *aprV5*Δ*C* strains by mass spectrometry.

Excised Gel band Size(kDa)	ProteinIdentification	Accession No matchedStrain	Coverage%^a^	BestMascot	Score^b^	Peptides matched by protein region/number of confirmed sequences^c^		
						Pro-PeptidesMS/MSMS	MaturePeptidesMS/MSMS	CTE PeptidesMS/MSMS
50	AprV2-CTE	A5EXI3	*aprV5*	49%	1060	2/0	10/6	4/3
50	BprV-CTE	A5EVD0	*aprV5*	34%	359	4/0	6/5	4/2
50	AprV2-CTE	A5EXI3	*aprV5*Δ*C1*	36%	530	0/0	8/7	5/2
50	BprV-CTE	A5EVD0	*aprV5*Δ*C1*	24%	53	0/0	5/1	3/1
50	AprV2-CTE	A5EXI3	*aprV5*Δ*C3*	40%	656	2/0	8/6	3/1
65	Pro-BprV-CTE	A5EVD0	*aprV5*	37%	905	3/3	6/5	5/2
65	Pro-AprV2-CTE	A5EXI3	*aprV5*	20%	144	2/1	3/0	3/0
65	Pro-BprV-CTE	A5EVD0	*aprV5*Δ*C1*	34%	390	3/1	7/6	3/2
65	Pro-BprV-CTE	A5EVD0	*aprV5*Δ*C3*	35%	386	3/1	7/6	4/2

a Coverage denotes the percentage of the full length protein sequence that has been matched to the MS data.

b Best MASCOT score obtained between analyses from different gel pieces excised from identical gels.

c MS value denotes the number of peptides matched to the MS data corresponding to sequences from within a specific region of the protein. MSMS value denotes the number of these peptides matches of which the sequences have been confirmed by MSMS analysis.

## Discussion

Virulent isolates of *D. nodosus* produce three homologous extracellular subtilases, AprV5, AprV2 and BprV, each of which is synthesized as a precursor molecule that is subsequently processed to form the mature active enzyme by an unknown mechanism. In this study, we have shown that AprV5 is responsible for its own maturation and for optimal processing of AprV2 and BprV. Our results have also revealed that the C-terminal extension of AprV5 is required for efficient processing of all three enzymes, presumably because it is required for the optimal processing of AprV5. In the absence of this domain, protease processing is delayed. Finally, we have provided evidence that the cleavage of the pro-domain and the C-terminal extensions of the AprV2 and BprV precursors occurs after secretion.

It has been reported that P-domain in some bacterial subtilases is required for both the folding and regulation of pH dependence of the catalytic domain [23], [34], [35]. In this study we have shown that AprV5 proteins lacking the C-terminal P-domain exhibited delayed production of the mature enzyme as a result of delayed pro-protein-C-terminal extension processing. This effect was not due to a secretion defect. We postulate that the C-terminal extension may function as an intramolecular chaperon that optimizes the folding of the precursor protein prior to proteolytic cleavage.

The processing of serine proteases involves autocatalytic cleavage of each of the pro- and C-terminal domains after membrane translocation [13], [36]; a similar process appears to occur for AprV5. By contrast, our results showed that the optimal maturation of AprV2 and BprV, including cleavage of the pro-region and the C-terminal extension, requires a functional AprV5 enzyme; processing was delayed in the absence of the P-domain of AprV5. There are precedents for these observations. In eukaryotes, subtilisin-like pro-protein convertases are responsible for the maturation of hormones and other proteins [37]. In bacteria, the subtilisin-like autotransporter protease SphB1 is essential for the maturation of the precursor of the secreted protein FhaB in *B. pertussis* [36]. We conclude that in *D. nodosus* the maturation of these proteases must occur very rapidly after secretion since the wild-type strain did not accumulate unprocessed forms of AprV2 and BprV at any growth stage and we could not detect a His-tagged C-terminal extension fragment in the *aprV5_His6_* derivative. The fact that we observed pro-mature-CTE and mature-CTE forms, but not pro-mature forms, of AprV2 and BprV in the *aprV5* mutants suggests that the C-terminal extensions of AprV2 and BprV are processed after pro-region cleavage, which provides further support for the hypothesis that the C-terminal extension plays an important role in the formation of the mature enzyme. It appears that AprV2 and BprV do not process each other or AprV5 since no unprocessed proteases were detected in the *aprV2* or *bprV* mutants, although an active band with a different electrophoretic mobility was observed in the *bprV* mutant, suggesting that BprV may have some role in protease processing.

Studies on the C-terminal extensions of other subtilases suggested that they formed a β-barrel structure that played a role in the protease secretion process [14], [16], [36], [38]. In agreement with these studies, it previously was suggested that AprV5, AprV2 and BprV were potential autotransporters, in which the C-terminal extension forms a β-barrel structure on the outer membrane to allow the proteases to be secreted [39]. However, our analysis indicates that their C-terminal extensions are not large enough to form a β-barrel structure. To determine if the C-terminal extension played a role in secretion, we constructed three separate C-terminal AprV5 deletion mutants. We found that there was no significant difference in the levels of AprV5 secreted into the culture supernatants in these mutant strains and the wild-type strain, indicating that the C-terminal extension was not essential for secretion. These results are in agreement with our previous finding that protease secretion involves the type IV fimbrial system [7], [8], [26].

Finally, previous studies provided evidence that the cleavage sites of the C-terminal extensions of AprV5 was at amino acid residues V477 [25]. By constructing strains expressing C-terminal truncated proteins of AprV5 we observed that the actual C-terminal cleavage site of AprV5 was different to that previously reported and is slightly closer to the C-terminus of AprV5, between residues 478 and 502.

In conclusion, we have demonstrated that the C-terminal extension of AprV5, which contains a P-domain similar to that of eukaryotic subtilisin-like proteases, is required for optimal processing and active enzyme production. We have also shown that AprV5 is responsible for the maturation of the precursors of AprV2 and BprV into their mature forms. These studies have made a major contribution to our understanding of how this pathogenic bacterium, which unusually produces three closely related subtilisin-like proteases, processes these proteases into their mature active forms.
